# The European response and transformation towards healthy energy

**DOI:** 10.1093/eurpub/ckac129.143

**Published:** 2022-10-25

**Authors:** V Matkovic, S Anne

**Affiliations:** Health & Environment Alliance HEAL, Brussels, Belgium

## Abstract

The EU Green Deal is the framework to achieve carbon neutrality by 2050, and decarbonisation and sustainability in the main economic sectors. This includes a revised greenhouse gas reductions goal of at least 55% for 2030, as well as commitments on zero pollution, healthier agricultural production, sustainable mobility and energy. Moreover, as part of the EU Green Deal, the European Commission has established a Just Transition Mechanism (JTM), to ensure that the transition towards a climate-neutral economy happens in a fair way, leaving no one behind. The aim is to mobilise at least €100 billion over the period 2021-2027 for carbon-intensive industries. But health costs of pollution are absent in Just Transition considerations. The Green Deal includes all the right “buzz words” and commitments for a healthy planet for healthy people. However, policy-makers also need to walk the talk when it comes to adopting the right laws and measures. The science and the acceleration of the climate change, environmental and health crises show us that we need to step up. HEAL believes a zero pollution- and health-based approach can be the overarching principle to deliver on these ambitions. The war in Ukraine as the latest crisis has brought up once again the need for the swift, without any delay, phase-out of all fossil fuels. In Europe, the recently proposed RePower EU plan aims to set a pathway for increased renewables, energy savings and diversification well before 2030. However, with a holistic view of the problem under a health lens, it is clear that in this transition from fossil fuels inequalities and social considerations need to be placed central. Of particular importance is to not give space for false solutions such as replacing Russian imports with fuels from other authoritarian regimes or economies which will only continue to perpetuate Europe's energy dependence, or to build up infrastructure that will continue the pollution (gas and biomass included).

